# Properties of WCCo Composites Produced by the SPS Method Intended for Cutting Tools for Machining of Wood-Based Materials

**DOI:** 10.3390/ma14102618

**Published:** 2021-05-17

**Authors:** Joanna Wachowicz, Tomasz Dembiczak, Grzegorz Stradomski, Zbigniew Bałaga, Marcin Dyner, Jacek Wilkowski

**Affiliations:** 1 Department of Mechanical Processing of Wood, Institute of Wood Sciences and Furniture, Warsaw University of Life Sciences, Nowoursynowska Street, 166, 02-787 Warsaw, Poland; jacek_wilkowski@sggw.edu.pl; 2 Faculty of Science and Technology, Jan Dlugosz University in Czestochowa, Armii Krajowej Street 13/15, 42-200 Czestochowa, Poland; t.dembiczak@ujd.edu.pl (T.D.); m.dyner@ujd.edu.pl (M.D.); 3 Faculty of Production Engineering and Materials Technology, Czestochowa University of Technology, Armii Krajowej Street, 19, 42-201 Czestochowa, Poland; gstradomski@wip.pcz.pl (G.S.); balaga.zbigniew@wip.pcz.pl (Z.B.)

**Keywords:** sintering, Spark Plasma Sintering, cemented carbides WCCo, powder metallurgy

## Abstract

This paper presents the possibility of using the Spark Plasma Sintering (SPS) method to obtain WCCo composite materials. Such materials are used as cutting blades for machining wood-based materials. Two series of composites, different in grain size and cobalt content, were analyzed in the paper. The produced materials were characterized using Scanning Electron Microscopy (SEM), X-ray diffraction (XRD), and tribological properties were determined. In addition, preliminary tests were carried out on the durability of the blades made of sintered WCCo composites while machining three-layer chipboard. The results of the microstructure analysis proved that the SPS method makes it possible to obtain solid composites. Phase analysis showed the occurrence of the following phases: WC, Co, and Co_3_W_9_C_4_. The lowest friction coefficient value was found in samples sintered using powder with an average primary particle size of 400 nm (ultrafine).

## 1. Introduction

WCCo (composite of tungsten carbide and cobalt) cemented carbides are widely used tool material but are still under research. They are characterized by high hardness and resistance to abrasive wear. However, these properties undergo changes depending on the cobalt content and the WC grain size. In recent decades, numerous research efforts have been undertaken to reduce the WC grain size to submicron and nanometer scales. Obtaining a fine-grained microstructure is important for the application of the materials. The use of nanometric WC grains results in better wear resistance of tools, compared to conventional WCCo carbide grades. To limit the grain growth process, so-called growth inhibitors, such as VC or Cr_3_C_2_, are added to the sintering process to retard the WC grain growth process [1,2,3,4,5,6,7,8,9].

In work [10], the kinetics of the WC grain growth process was analyzed and the evolution of WC grain morphology during annealing was studied by high-resolution scanning electron microscopy. The results showed that the grain growth process consists of an initial stage of rapid growth which normally occurs during heating and normal grain growth during isothermal holding. Rapid grain growth is a problem that affects not only sintered tungsten carbide but also the fabrication of a wide range of other nanocrystalline materials. Compared to sintering of conventional micron-sized powders, sintering of nanometer-sized powders involves the additional challenge of maintaining grain sizes at the nanoscale after full compaction has been achieved.

According to the current knowledge, various powder metallurgy techniques are used to produce WCCo composites. These include free sintering, Hot Isostatic Pressing (HIP), Hot Pressing (HP). However, these methods require high temperatures and long sintering times, which translates not only into properties but also into production costs. The need to obtain materials with enhanced properties requires the use of innovative, unconventional sintering techniques. These include current-activated methods—the so-called FAST techniques (Field Assisted Sintering Technology). Among these methods, the SPS (Spark Plasma Sintering) method is the most widespread. This technology allows for the obtaining of a wide range of materials [11,12,13,14,15,16,17,18,19,20,21,22,23,24,25,26].

In the SPS method, the powder is placed in a graphite set (die + 2 punches) which forms a kind of heating element when an electric current is passed through it. The entire set is subjected to a uniaxial pressure during the sintering process. The powder is heated by the Joule heat generated when pulses of electric current flow through the die and the sintered powder. The temperature during the process is measured by means of a thermocouple placed in an electrode or a pyrometer. The SPS process takes place using a high intensity but low voltage (of a few volts) electrical discharges. In theory, each newly produced impulse should flow along a different path between the powder particles. The effect of plastic flowing of the material occurs due to the pressure applied between the punches. The combination of this phenomenon with diffusion processes makes it possible to obtain a material with a porosity of less than 1%.

In the SPS process, their surface is more cleaned and activated, compared to conventional sintering methods due to the pulses of direct current flowing through the powder particles. Therefore, high-quality sinters are obtained at a lower temperature and in a shorter time. As a result of the flow of electrical impulses, there is a temporary local increase in temperature of up to several hundred degrees Celsius. Joule heat is generated, which is dispersed on the particle surface. This causes evaporation and melting on the surface of the powder particles and the formation of a bond, or so-called “neck”, between the particles [27,28,29,30,31].

The rapid development of the furniture market results in the need to produce tools with increasingly better properties which makes it possible to increase the efficiency of the production. One of the prospective paths of development for blades intended for cutting wood-based materials is carbides made using the Spark Plasma Sintering method. In terms of machining, particleboard is a very demanding material. It is characterized by different densities on the cross-section. The individual components of the boards differ in their machinability and density. Moreover, there are mineral impurities, which are the main material factor in the wear of cutting tools. Additionally, it is a material with poor heat-conducting properties which cannot be machined with liquid coolant because of the hygroscopic properties of the lignocellulosic particles. The cutting edges of the blades working in the machining process are exposed to very high temperatures and thus to rapid wear [32,33].

Wood-based materials require different processing techniques, compared to metals. There are no following wear processes observed during machining of wood-based materials: adhesive wear, diffusion wear, and direct oxidation. This is due to too low a temperature at the cutting edge, differences in physical and chemical properties of the workpiece material and the blade material, and too low a pressure, which results in no increase in the coefficient of friction with progressive blade wear, respectively. However, other phenomena related to the moisture content of the workpiece material and aggressive chemicals present in it may be noted, these are high-temperature tribological phenomena, low-temperature tribological phenomena (low material moisture content), electrostatic discharge (very low material moisture content). During machining, the machining tools wear and tear and as a result, the following changes take place: dimensions, the shape of the cutting edge, tool mass, chemical, and physical properties of the surface layer of the blade. This results in a decrease in the machining properties of the blade and a change in the physical and chemical processes. The loss of cutting properties can be caused by several phenomena: wear, chipping, splitting, breaking, or cracking. They may also occur simultaneously. The partial effects which make up the tool wear process are mechanical, thermal, chemical, and physical interactions between the tool and the material being cut in the environment defined by the machine tool [32,33,34,35].

This study aimed to determine tribological properties and the degree of wear of SPS tools compared to commercially available blades (of similar chemical composition). On the basis of the obtained results, it was supposed to determine the perspective of using the tools made by the SPS method in the furniture industry.

## 2. Materials and Methods

**Figure 1:**
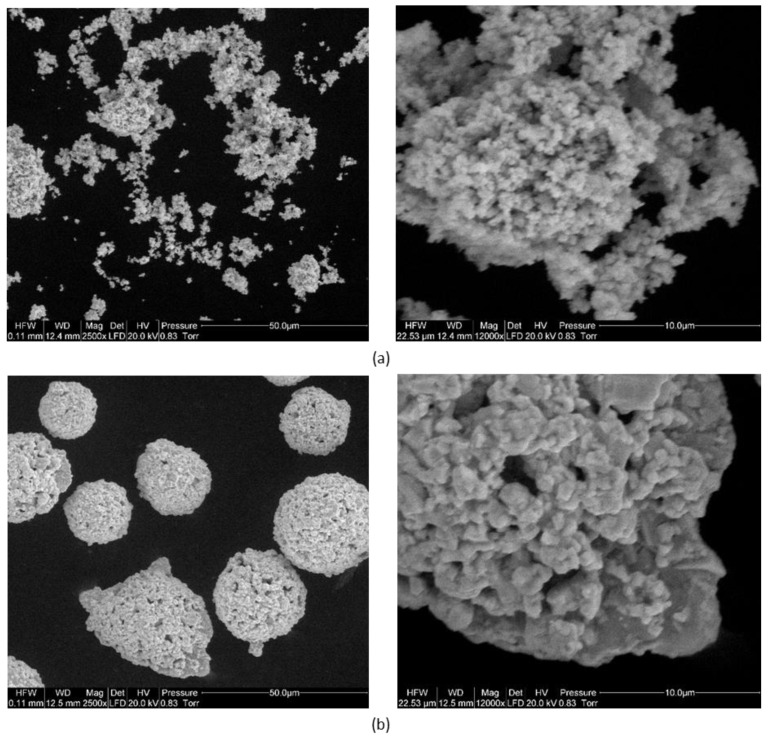
SEM images of powders used to prepare sinters: (**a**) primary particle size of WC about 1 μm (submicron); (**b**) primary particle size of WC approx. 400 nm (ultrafine).

Mixed WC and Co powders were used in the tests. The powders used differed in the size of the primary WC particles. The particle size of one of them was ca. 1 μm (submicron), while that of the other was ca. 400 nm (ultrafine). Microscopic observations (using SEM–FEI QUANTA 200, FEI, Hillsboro, OR, USA) of powder with a particle size of 400 nm showed spherical agglomerates of about 20 µm. In contrast, the WC powder with a particle size of about 1 µm was characterized by an irregular shape, with only some powder particles forming irregular agglomerates of different sizes (Figure 1).

**Figure 2:**
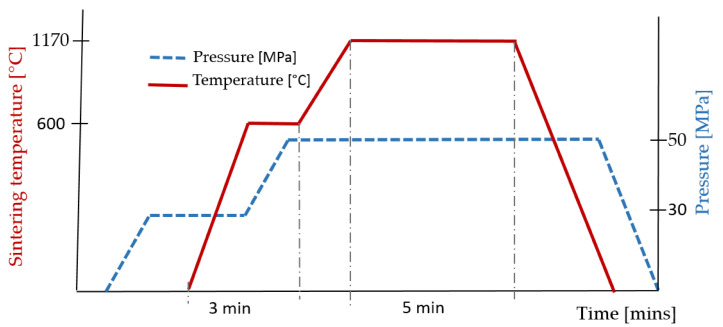
SPS cycle.

The WCCo carbide sintering process was carried out using a Spark Plasma Sintering (SPS, prototype device, Łukasiewicz Research Network - Institute of Electronic Materials Technology, Warsaw, Poland device, in two stages. The degassing stage was carried out at a temperature of about 600 °C for 3 min, increasing the uniaxial pressure from 30 to 50 MPa in the second minute. In the second stage, the sintering itself was carried out at 1170 °C for 5 min (Figure 2).

The density of samples was measured using the Archimedes method, with a ME204 balance (Mettler Toledo, Greifensee, Switzerland). Hardness measurements were carried out using the Vickers method in accordance with the PN-EN ISO 6507-1: 2007 standard using an FM-700 hardness tester (Future Tech) at a load of 294.2 N. In addition, an important parameter from the point of view of failure resistance was determined, i.e., resistance to brittle fracture (critical stress intensity factor KIC). Measurements of the brittle fracture length were carried out with a Nikon Ma200 optical microscope using the Nis-Elements D software (Ma200, Nikon, Tokio, Japan). The resistance to brittle fracture (critical stress intensity factor *K_IC_*) was determined using Equation (1), where *HV*30 is the hardness measured under a load of 294.2 N, Σ*l*—the sum of the lengths of cracks formed in the corners of the imprint.

(1) $$K_{IC}=0.15\cdot \sqrt{\frac{HV30}{\sum l}}$$

Observations of the microstructure of the composites obtained were carried out using SEM–scanning electron microscope—FEI QUANTA 200. Phase composition analysis was performed by X-ray diffraction with a SEIFERT XRD 3003 T-T X-ray diffractometer (Rich. Seifert & Co. Röntgenwertk, Ahrensburg, Germany) using cobalt anode lamp radiation with a wavelength of λCoKα = 0.17902 nm.

Tribological wear of WCCo sinters was carried out using a T-05 device. The test set-up consisted of a stationary specimen, made of the test material, pressed with a set force of 5 N, 10 N, and 20 N against a roller rotating at a set speed of 101.92 rpm in one direction.

The tribological wear test consisted of twenty cycles, each lasting 10 min. After twenty cycles, the friction force was measured for each sample to determine the coefficient of friction. Each sample was cleaned with an ultrasonic cleaner before and after each cycle, then dried and weighed with an accuracy of 0.00001 g to determine the mass loss.

**Figure 3:**
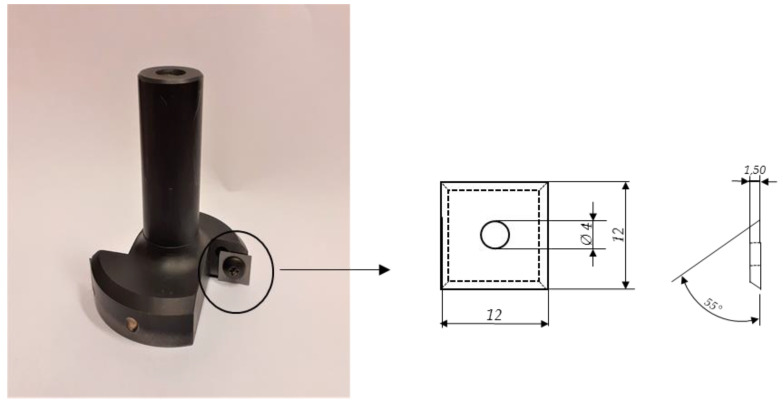
Two-blade cutter.

Two groups of WCCo tools manufactured using the SPS method and commercial tools of similar composition and properties were used in the tests. Durability tests were conducted on blades, with four cutting edges, measuring 12 × 12 × 1.5 mm and a blade angle of 55° (Figure 3).

**Figure 4:**
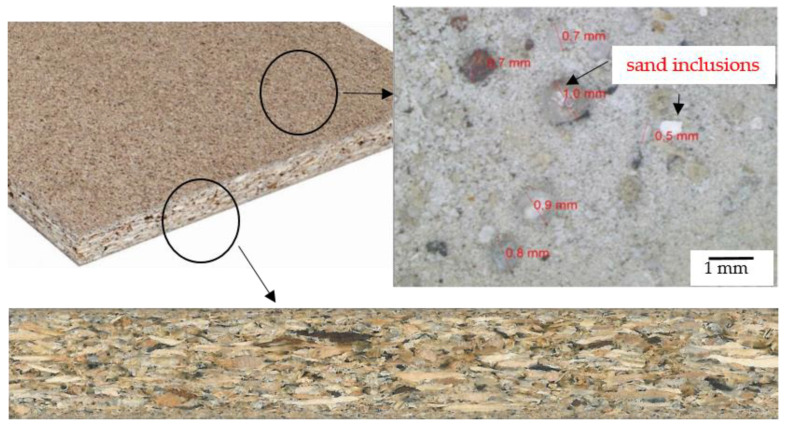
Three-layer chipboard.

Raw three-layer chipboard (Figure 4) with a thickness of 18 mm was used to carry out the tests. Selected properties of the chipboard are summarized in Table 1.

Blade stability tests were carried out on a Busselato Jet 130 machining center, with the blades placed in a Faba double-edged burr model FTS.07P6041.06. The cutting process was carried out to a depth of 6 mm at a spindle speed of 18,000 rpm, with a feed per tooth of 0.15 mm and a feed speed of 2.7 m/min. The abrasion of the contact surface was measured every 1 m of feed distance using a bench microscope until the critical value of VBmax = 0.2 mm was reached. Wear and tear were measured using a shop microscope.

## 3. Results and Discussion

**Figure 5:**
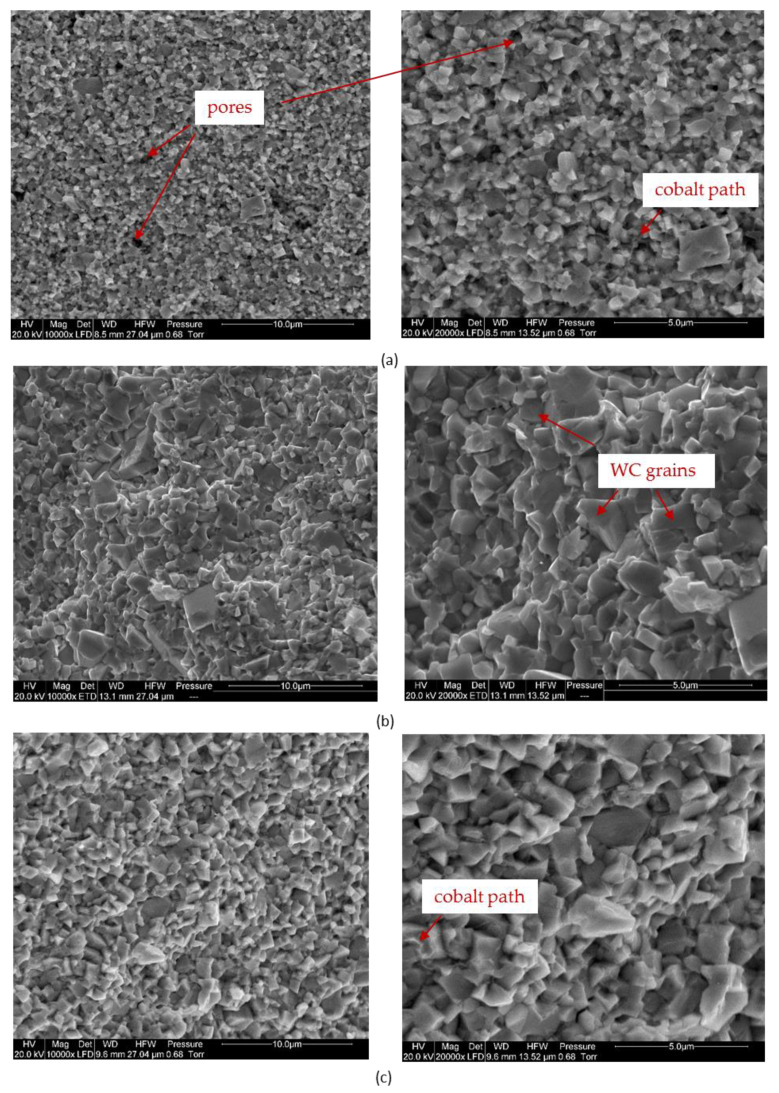
Microstructures of the composites: (**a**) submicron, (**b**) ultrafine, (**c**) commercial.

**Figure 6:**
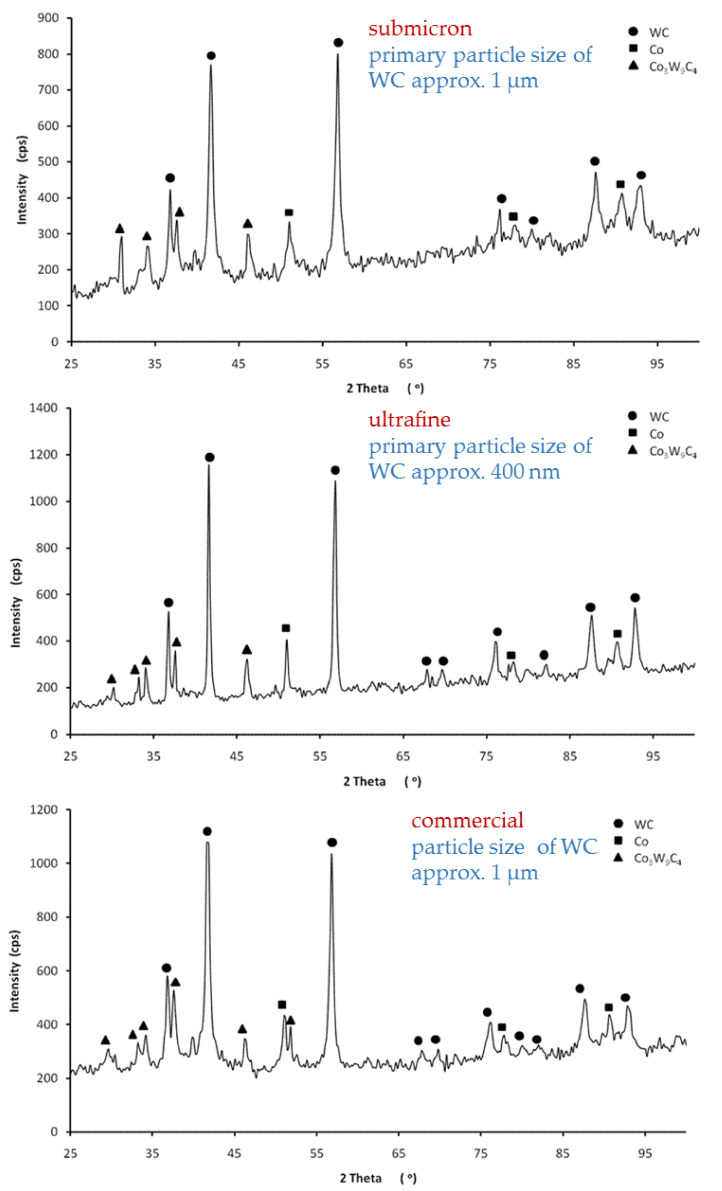
Diffraction analysis of the composites studied.

**Figure 7:**
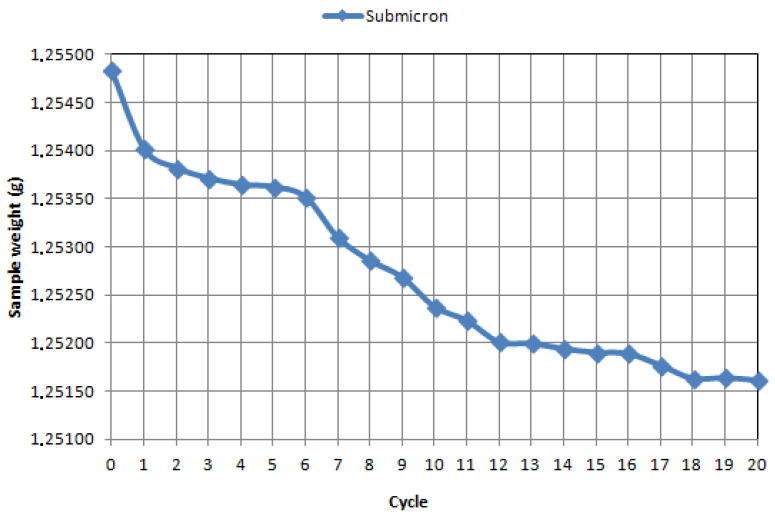
Loss of mass during tribological tests after 20 cycles for sintered micro-powder at 5 N load.

All samples intended for further testing, manufactured from powders had densities close to the theoretical density value (above 99% of the theoretical density). The hardness results and critical stress intensity factor K_IC_ values are summarized in Table 2 and Figure 5, Figure 6 and Figure 7. The hardness results obtained had similar values over the entire cross-section of the samples. This indicates uniform sintering of the samples in the entire volume. The maximum value of the stress intensity factor was obtained for commercial samples.

Figure 5 shows the microstructures of the fractures of composites, obtained after the sintering process and a commercial sample with a similar chemical composition.

The microstructure of the fractures of submicron composites is characterized by well-formed WC grains. During the fracture of the sample, a crack occurred at the grain boundary and not through the center of the grains. The SEM images of WCCo composites show a slight porosity. Uniformly distributed cobalt can be observed along the WC grain boundaries. Composites made of ultrafine powder have a compact sinter structure over the entire cross-section of the sample. No pores are observed and the WC grains have characteristic sharp edges.

During the sintering process in general it’s not easy to control grain size. This can be referred to as plastic hot deformation of metals, and recrystallization process. As for the SPS, the technique is contrary to other technologies that give the possibility to work with the current parameters. The combination of impulse duration interval combination of time, temperature, and strains gives more possibilities of impact. As for the grain size, the fracture toughness and hardness are combined. The fracture toughness in general with the smaller grain, the larger/higher it is. But for sintered composites, these parameters have an influence also porosity and diffusion bonding. Therefore, even materials with very similar grain sizes made with different techniques can have very different mechanical properties. Porosity also influences the hardness. The hardness of submicron and commercial be taken as very similar. No pores were observed at the microstructure of the commercial samples. The sintering process of these materials was carried out using a traditional method involving the liquid phase. This is evidenced by, among others, cobalt, which is evenly distributed along the grain boundaries of the WC, as shown in the SEM photos of the microstructure of the received composites. The presence of a liquid phase introduces many changes in the course of powder densification processes and has a decisive influence on the course of sintering (densification).

Tungsten carbide (WC) and cobalt (Co) were found to be present in all the samples tested. Diffraction analysis showed the occurrence of a regular high-temperature variety of cobalt phases, which is the bonding phase in the WCCo composite. This allows us to conclude that the occurred cobalt phase is a solid solution containing dissolved carbon and tungsten, which stabilizes the high-temperature variety of cobalt. Moreover, as a result of reactions that occurred during the sintering process of the samples, the Co_3_W_9_C_4_ phase was found to be present in the structure (Figure 6).

In work [36], it was shown that during reactive sintering of mixtures of W-6% Co-C powders, the reaction product between these elements depends on the relative carbon and cobalt contents. At a carbon content of 7 wt%, WC carbide forms by itself, and densification occurs in the presence of a cobalt-based liquid phase. In contrast, at lower carbon contents, W_2_C carbide forms first and reacts with cobalt to form mixed Co_3_W_9_C_4_ carbide. The hardness of the material is increased, but the amount of liquid phase is reduced and the sintering densification is limited. In the presence of mixed carbide, the cobalt content should be sufficient to produce the amount of liquid phase required for sintering densification. Therefore, the choice of the initial carbon content allows influencing the type of phases obtained and the densification conditions of sintering in the liquid phase.

The presence of intermediate carbide Co_3_W_9_C_4_ in the material when cemented carbides with low cobalt contents were also observed in works [37,38]. Co_3_W_9_C_4_ phase is formed as a result of the reaction of W_2_C and Co_3_W_3_C, according to Equation (2).

Co_3_W_3_C + 3W_2_C → Co_3_W_9_C_4_, (2)

The conventional sintering process, which is carried out with the cobalt liquid phase, favors the WC grain growth. Traditionally, the sintering process is carried out in two stages, with pre-sintering. Initially, only the diffusion of C and WC in γ-Co takes place in the temperature range 600–1250 °C. Then, at higher temperatures, cobalt dissolves some WC into the solid-state. A further increase in temperature causes the cobalt to melt and as the sintering progresses, WC is dissolved in it. During slow cooling, the dissolved tungsten carbide is released from the γ-phase, causing grain growth. In the SPS method, the occurrence of the cobalt liquid phase is also possible, which is related to the specific heating conditions. During the current flow, the powder is heated to a high temperature and after the current disappears, it cools very rapidly to the set sintering temperature. This promotes the formation of necks between the particles. A very intensive transport of material into the neck area is then observed. The electrical discharges which occur cause a locally significant temperature rise [39,40]. When the current flow ceases, it is rapidly reduced to the set sintering temperature. Thus, consolidation by the SPS method is also carried out in the presence of a cobalt liquid phase. This is evidenced by the uniform distribution of cobalt across the WC grain boundaries. If the process is carried out without the participation of the liquid phase, the cobalt occurs in the form of agglomerates. In the SPS process, due to the very short duration of the high temperature and its rapid decrease, grain growth is strongly limited. The tungsten and carbon dissolved in the cobalt did not manage to separate from it.

Tribological tests, as already mentioned, were carried out in the roll-block system. This choice of test method was dictated by the fact that this technique more closely replicates the operating conditions of the materials studied. Table 3 presents the parameters and results of tribological wear tests for the sinter produced.

The recorded friction forces for the roller pad system allowed to determine the coefficient of friction for the WCCo sinters tested, as presented in Table 3.

The coefficient of friction is equal to the ratio of the friction force *T* to the forces *Fn* of the body on the ground.

(3) $$\mu =\frac{T}{F_{n}}$$

where: *T*—recorded friction force during tests [N], *Fn*—set pressure force [N].

Based on the obtained test results, it was found that for a contact force of 5 N the recorded friction force values decrease. Averaged values of the friction coefficient were determined from the entire range of the friction path at μavg = 0.13 for the WCCo sinter produced from submicron by SPS (Spark Plasma Sintering). Based on tribological tests, it was found that a lower coefficient of friction was obtained for the WCCo sinter produced from ultrafine powder using the SPS (Spark Plasma Sintering) method, which was μavg = 0.100. For commercial WCCo cutting inserts produced using the conventional method, the average coefficient of friction was μavg = 0.151. Based on the obtained average values of the friction coefficient at a load of 5 N, significant dynamics of changes in the friction coefficient value were found. This is due to different sizes of powder particles (ultrafine, submicron) and the obtained mechanical properties after sintering at a temperature of 1170 °C and an applied pressure of 50 MPa.

At increased values of pressing force of 10 and 20 N of the friction couple, the average friction coefficients for the produced WCCo composites show a definite tendency to increase and the courses of their changes as a function of the friction path are stable and less sensitive to slip velocities.

**Figure 8:**
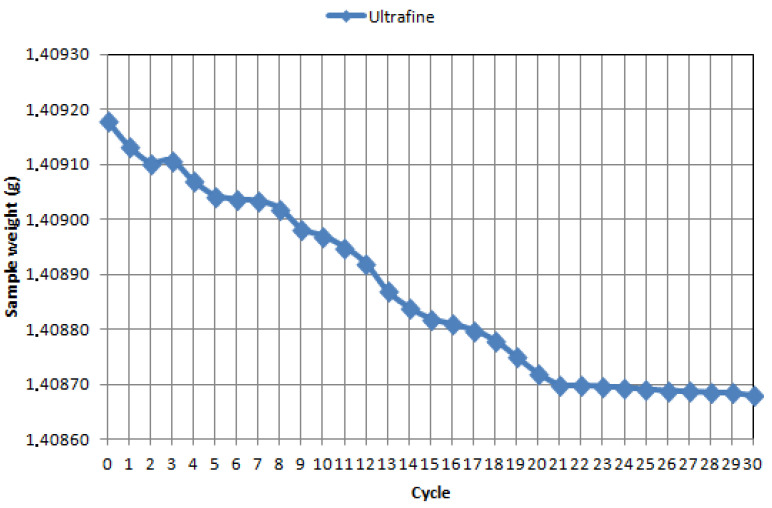
Loss of mass during tribological tests after 30 cycles for sintered WCCo made of ultrafine with 5 N load.

**Figure 9:**
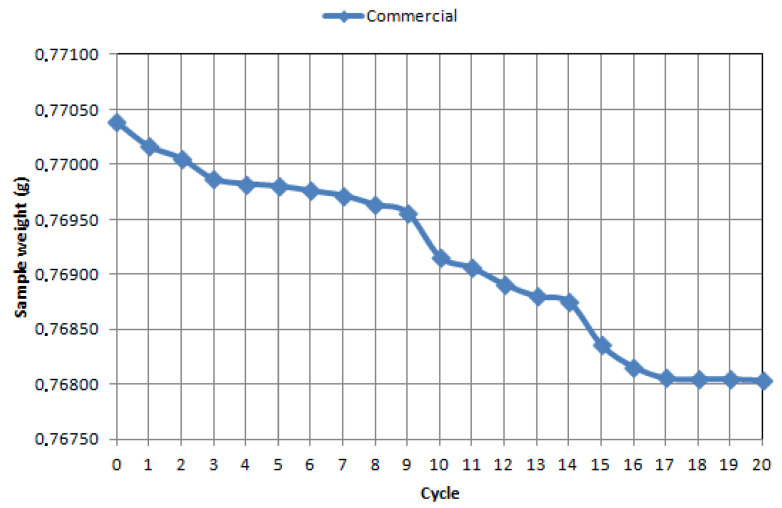
Loss of mass during tribological testing after 20 cycles for a commercial WCCo sintered cutting insert at 5 N.

Tests of friction coefficient and wear index have shown that WCCo sinters made from ultrafine and submicron powders have a favorable effect on tribological properties compared to commercial cutting inserts. As shown in Table 3, the reduction of frictional forces and, consequently, the reduction of energy lost in frictional contact resulted in a significant decrease in the wear index of the WCCo sinters studied. From the point of view of the use of sinters tested on cutting tools, a decrease in the friction coefficient should lead to a decrease in the temperature of the working tool and thus result in a slowing down of its wear processes. Figure 7, Figure 8 and Figure 9 present the wear curves obtained for individual samples.

On the basis of the mass loss analysis, it was proved that the WCCo sinter produced from submicron powder has a slow stabilized abrasive wear, it was found that the total wear occurs after the seventeenth test cycle (10,200 s). The WCCo sinter produced from ultrafine powder shows stabilized wear, while it was found that abrasive wear occurs after the twentieth cycle (12,000 s).

In contrast, commercial WCCo cutting inserts are characterized by stabilized wear after the 16th cycle. It should also be noted that the nature of the inclination of the curves indicates different wear characteristics of each material. For the ultrafine material, the first wear stop is already visible after cycle 2, further on in cycles 4–6, and then only in cycle 20. The submicron material is more similar in nature to the commercial material; stabilization zones are observed in similar cycles and in similar interval lengths. The material made of nano-powder stabilizes the longest, but it should be assumed on the basis of the obtained curves that the period of stable wear will be the longest for it.

Wood-based materials are characterized by heterogeneous structure, complex chemical composition, and anisotropy of structure and therefore require different processing techniques, compared to metals. In addition, they show low thermal conductivity, which causes a significant increase in tool wear and tear.

**Figure 10:**
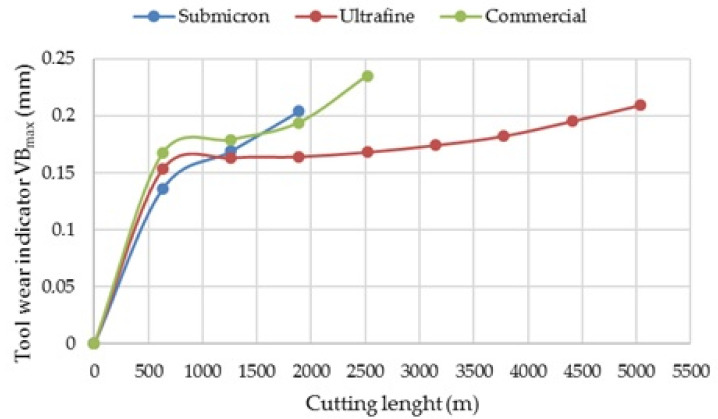
Example wear curves of the tested blades.

Example wear curves of the tested multi-edged knives are presented in Figure 10. The obtained curves have a course similar to the Lorentz wear curve, with the difference that after obtaining a wear criterion of 0.2 mm, the tool was withdrawn from service. However, the curves are characterized by the occurrence of two separate periods in which the wear intensity changes (the running-in period and the wear area in the steady-state). The longest courses of wear curves were determined for tools obtained from ultrafine powder, in which the size of the original particles was 400 nm. The use of ultrafine insert did not change the character of the wearing course, e.g., by eliminating the running-in phase, but it caused the normal wear time (second phase) to be extended more than twice. Based on Figure 10, it was found that the use of ultrafine carbide blades increases the service life significantly compared to submicron and commercial carbide blades. The results obtained are consistent with literature studies.

The presented curves are characterized by the occurrence of two distinct periods in which the intensity of wear changes. The first period is characterized by the initially large but then decreasing wear intensity (Breakin period). It is followed by a period characterized by a constant intensity of wear typical for normal operation (Steady-state wear region). The Break-in period was dominated by chipping and breaking off of the cutting edge (chipping, microchipping), which was related to the mechanisms of erosion, fatigue resistance. The intensity of these wear forms is largely random. In the steady-state wear phase, the abrasive mechanism undoubtedly dominated in the form of microcutting, furrowing, scratching, cracking, and chipping of WC grains. The occurrence of such tool wear mechanisms was also confirmed in other studies [41,42].

**Figure 11:**
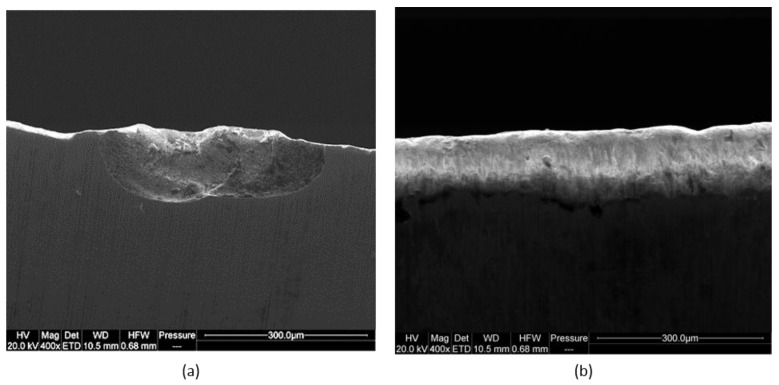
Wear types of tested blades (**a**) chipping (**b**) abrasion.

Figure 11 presents the wear patterns of blades during chipboard machining. Two types of cutting-edge wear dominated the tested blades: crumblings (chipping, micro-chipping) and through abrasion. The resulting chipping of the cutting edge is the result of exceeding the permissible mechanical tensile stresses. Numerous sand inclusions in the workpiece material may be the cause of this type of stress. The results showed that no thermal wear of the tools or as a consequence of adhesion was observed in the case of chipboard machining.

## 4. Conclusions

The following conclusions have been drawn on the basis of the tests carried out: the applied SPS sintering method allows the use of dense composites (relative density >99%), using powders with different gradations of primary particles (around 1 µm and around 400 nm).

All the composites obtained were characterized by a homogeneous microstructure. The relatively low sintering temperature did not cause a discernible increase in WC grain size when sintering powders with an average primary particle size of about 1 µm.

Results obtained in cutting tests of a three-layer chipboard confirmed the difficult machinability of this material and the influence of the granularity of the original powder mixture on blade life. The application of the new sintering method resulted in an increase in tool life for blades obtained from powder with a primary particle size of 400 nm. Microscopic analyses of the blade after operation indicated wear through abrasion and chipping.

Blades sintered using ultrafine powder are characterized, in comparison with conventional blades, by more than doubled tool life in the process of chipboard cutting for an assumed value of blunt index VB = 0.2 mm.

In further tests, it would be interesting to analyze blades with a different composition of chemical composition and the use of other modified sintering conditions.

## Figures and Tables

**Table 1:** Selected properties of the tested three-layer chipboard.

Wood-Based Board	Density [kg/m^3^]	Brinell Hardness	Bending Strength [%]	Elasticity Module [MPa]	Sand Content [%]
Three-layer chipboard	648	2.6	8.7	2212	0.185

**Table 2:** Basic properties of the tested materials.

Experimental Materials	Apparent Density (g/cm^−3^)	Relative Density (%)	Hardness HV30	Fracture Toughness (MPa/m ½)
Submicron (sintering)	14.74	99.26	1736 ± 38	11.3
Ultrafine (sintering)	15.20	99.99	1622 ± 40	12.5
Commercial	15.20	100.00	1705 ± 40	26.6

**Table 3:** Tribological and wear properties of investigated materials.

Experimental Materials	Applied Load (N)	Distance (m)	COF (-)	Wear Rate (%)
Submicron	5	110	0.130	0.256
	10	110	0.221	0.435
	20	110	0.397	0.781
Ultrafine	5	110	0.100	0.035
	10	110	0.175	0.061
	20	110	0.323	0.112
Commercial	5	110	0.151	0.304
	10	110	0.277	0.557
	20	110	0.541	1.088

## Data Availability

Data sharing is not applicable to this article.
